# A Remarkable Man

**Published:** 1898-02

**Authors:** 


### A Remarkable Man.

Most of those who have been accustomed to attend the meetings of the American Dental Association will remember one who is usually present, and especially when the meetings are held in the eastern part of the country—this is DR. JESSE C. GREEN, of West Chester, Penn.

Dr. Green is seventy-six years of age. This is not very remarkable however, for a good many in our profession have attained this longevity and a few are older. He was descended from English ancestry, and he is not very peculiar in this respect, for a good many are in like condition.

For more than two hundred years Dr. Green's ancestors have been members of the Society of Friends, and to that society the doctor has always adhered.

His early life was spent on a farm (a good place to be reared) where he resided until 1841.

He was engaged in teaching from 1836 to 1842. In 1842 he turned his attention to the study of dentistry, and began his practice in April, 1843, in West Chester, Penn., where he has remained in practice ever since. The degree of D.D.S. was conferred upon him by the Pennsylvania College of Dental Surgery of Philadelphia.

The doctor has been remarkably successful in the practice of his profession. He possesses a combination of faculties that insure success. He possesses rare aptness in mechanics, was of studious habit, energetic and industrious. He possesses unwavering energy, and has attained success in life without making a compromise with the spirit of unfairness, deceit or untruth in any form. Perhaps the most remarkable thing concerning Dr. Green is the maintenance of his mental and physical faculties. His mind is as clear to-day as it has ever been. His physical strength and ability seem scarcely if at all impaired by age. He pursues the practice of his profession with the same earnestness, interest and efficiency as in former days. His step and movements have scarcely lost any of the original vigor and elasticity. He has retained almost perfect health throughout his past life. This he has done because he so strictly obeyed the laws of life, health and correct living.

In this respect Dr. Green is an illustrious example that is worthy of the emulation of every member of our profession. Especially is the career of such as he worthy of the emulation of every young man in our profession, and a good many who are well on in life's road will do well to follow such an example.

